# Methamphetamine use and malnutrition among street-involved youth

**DOI:** 10.1186/1477-7517-7-5

**Published:** 2010-03-08

**Authors:** Dan Werb, Thomas Kerr, Ruth Zhang, Julio SG Montaner, Evan Wood

**Affiliations:** 1 British Columbia Centre for Excellence in HIV/AIDS, St Paul's Hospital, Vancouver, Canada; 2 Department of Medicine, Faculty of Medicine, University of British Columbia, Vancouver, Canada

## Abstract

We sought to explore the effect of crystal methamphetamine use on the risk of experiencing malnutrition among street-involved youth in Vancouver, Canada. Risk of malnutrition was defined as being hungry but not having enough money to buy food. Socio-demographic and drug use factors associated with risk of malnutrition were investigated using univariate and multivariate analysis among a prospective cohort of street-involved youth known as the At-Risk Youth Study (ARYS). Between September 2005 and December 2006, 509 street-involved youth were enrolled in ARYS, among whom 21% reported being at risk of malnutrition as defined above in the previous six months. In multivariate analysis, only non-injection crystal methamphetamine was significantly associated with being at risk of malnutrition among this cohort (Adjusted Odds Ratio [AOR] = 1.60, 95% Confidence Interval [CI]: 1.03 - 2.48, *p *= 0.036). Interventions seeking to address food insecurity among street youth may benefit from considering drug use patterns since methamphetamine use predicted higher risk in this setting.

## Findings

Over the last decade, crystal methamphetamine use has emerged as a unique and significant public health concern, and data on the prevalence of crystal methamphetamine use have shown that its use is increasing in North America, particularly among young gay men and young injection drug users [1,2]. Studies have also reported that crystal methamphetamine use is associated with a variety of physiological and neurological disorders [3], as well as with a number of risk behaviours for HIV transmission, and that these risks are heightened among street-involved youth [2]. This is of concern given that the health of street-involved youth is already often compromised by widespread unstable housing and chronic food insecurity [4].

Little research, however, has been conducted on the association between patterns of drug use and food security among youth. Specifically, knowledge gaps exist concerning the potential unique impact of crystal methamphetamine use on risks of malnutrition among street-involved youth populations. We therefore sought to explore the effect of crystal methamphetamine use on the risk of experiencing malnutrition among a cohort of street-involved youth in Vancouver, Canada.

We evaluated factors associated with malnutrition among participants enrolled in the At-Risk Youth Study (ARYS), a prospective cohort of street-involved youth aged 14 to 26 in Vancouver, Canada, which has been described in detail previously [5]. In brief, at baseline and semi-annually, ARYS participants complete an interviewer-administered questionnaire and provide blood samples for diagnostic testing. In the present study, Pearson's Chi-square test and multivariate analysis were used to determine factors associated with ever having experienced malnutrition among this cohort.

Our primary independent variable of interest was crystal methamphetamine use, though we accounted for a wide array of socio-demographic, drug use and behavioural variables, all of which are shown in Table 1. All variable definitions were identical to earlier reports from our setting [6] and all behavioural and drug use variables refer to behaviours in the previous six months. We defined the dependent variable based on responses to the following ARYS survey question: "I am often hungry but I don't eat because I can't afford enough food". Respondents answering "Often true" or "Sometimes true" were defined as being at risk of malnutrition; those answering "Never true" were defined as not being at risk of malnutrition. All tests were two-tailed and the significance level was set at *p *< 0.05. All statistical analyses were performed using SAS software version 9.0 (SAS, Cary, NC).

**Table 1:** Univariate analysis of factors associated with malnutrition among a cohort of street-involved youth (*n *= 509)

	Being at risk of malnutrition in the last six months					
					
Characteristic*	No (*n *= 404)	Yes (*n *= 105)	Total	OR	95% CI	*p *value
**Age**						
Median (and IQR)	21.8 (19.8 - 23.7)	22.7 (20.7 - 24.3)		1.10	(1.01 - 1.19)	0.024
**Gender**						
Male	284 (70.3)	76 (72.4)	360			
Female	120 (29.7)	29 (27.6)	149	0.90	(0.56 - 1.46)	0.676
**Caucasian ethnicity**						
No	129 (31.2)	25 (23.8)	154			
Yes	275 (68.1)	80 (76.2)	355	1.50	(0.91 - 2.46)	0.107
**Injecting in public**						
No	318 (78.7)	74 (70.5)	392			
Yes	86 (21.3)	31 (29.5)	117	1.55	(0.96 - 2.51)	0.074
**Requiring help injecting**						
No	378 (93.6)	97 (92.4)	475			
Yes	26 (6.4)	8 (7.6)	34	1.20	(0.53 - 2.73)	0.665
**Non-injection crystal methamphetamine use**						
No	237 (58.7)	49 (46.7)	286			
Yes	167 (41.3)	56 (53.3)	223	1.62	(1.05 - 2.49)	0.027
**Crack use**						
No	180 (44.5)	34 (32.4)	214			
Yes	224 (55.5)	71 (67.6)	295	1.68	(1.07 - 2.64)	0.024
**Injection crystal methamphetamine use**						
No	334 (82.7)	78 (74.3)	412			
Yes	70 (17.3)	27 (25.7)	97	1.65	(0.99 - 2.75)	0.051
**Injection heroin use**						
No	330 (81.7)	76 (72.4)	406			
Yes	74 (18.3)	29 (27.6)	103	1.70	(1.04 - 2.80)	0.035
**Injection cocaine use**						
No	364 (90.1)	96 (91.4)	460			
Yes	40 (9.9)	9 (8.6)	49	0.85	(0.40 - 1.82)	0.681
**Non-injection opiate use**						
No	382 (94.6)	96 (91.4)	478			
Yes	22 (5.5)	9 (8.6)	31	1.63	(0.73 - 3.65)	0.233

Note: CI = Confidence Interval; IQR = Interquartile Range

*All drug use variables were defined as ever in the last six months.

In total, 509 youth were recruited into the ARYS study between September 2005 and December 2006. Among this cohort, 149 (29%) were women, 154 (30%) were non-Caucasian, and the median age of participants was 22 years (Interquartile Range [IQR]: 20.0 - 23.9). Overall, 105 (21%) individuals reported being at risk of malnutrition in the previous six months.

As shown in Table 1, in univariate analyses, factors positively associated with being at risk of malnutrition included age (Odds Ratio [OR] = 1.10, 95% confidence interval [CI]: 1.01 - 1.19, *p *= 0.024), non-injection crystal methamphetamine use (OR = 1.62, 95% CI: 1.05 - 2.49, *p *= 0.027), crack use (OR = 1.68, 95% CI: 1.07 - 2.64, *p *= 0.024) and injection heroin use (OR = 1.70, 95% CI: 1.04 - 2.80, *p *= 0.035). Those variables that were found to be non-significant in univariate analyses (all *p *> 0.05) are also shown in Table 1.

As shown in Table 2, in multivariate analysis, only non-injection crystal methamphetamine use was associated with risk of malnutrition (Adjusted Odds Ratio [AOR] = 1.60, 95% CI: 1.03 - 2.48, *p *= 0.036) after adjustment for all other variables found to be significantly associated with being at risk of malnutrition in univariate analyses.

**Table 2:** Multivariate analysis of factors associated with being at risk of malnutrition among a cohort of street-involved youth (*n *= 509)

Characteristic*	Adjusted Odds Ratio (95% CI)	*p *value
**Non-injection crystal** **methamphetamine use**		
Yes *vs*. No	1.60 (1.03 - 2.48)	0.036
**Age**		
≤ 22 years *vs*. > 22 years old	1.07 (0.99 - 1.17)	0.097
**Crack use**		
Yes *vs*. No	1.44 (0.90 - 2.31)	0.125
**Injection heroin use**		
Yes *vs*. No	1.41 (0.84 - 2.36)	0.193

Note: CI = Confidence Interval

*All drug use variables were defined as ever in the last six months.

In the present study, over 20% of participants reported being at risk of malnutrition in the prior six months as defined as often being hungry but not having enough money to buy food. In multivariate analysis, and despite intensive adjustment for a range of drug use, behavioural and socio-demographic factors, only non-injection crystal methamphetamine use was independently associated with being at risk of malnutrition.

These findings may reflect a unique risk of malnutrition associated with crystal methamphetamine use among street-involved youth. While past studies on crystal methamphetamine use among street-involved and gay male youth populations have reported an association between use of this drug and risk behaviours associated with the transmission of HIV and other blood-borne diseases [2,7], we believe our study is the first to identify crystal methamphetamine use as a potential determinant of malnutrition. Our findings indicate that interventions focussed primarily on the risk of HIV transmission and the neuropsychological effects of use of this drug among youth [8] may need to be re-evaluated in order address the broader impact of crystal methamphetamine use on a wider variety of health determinants, such as malnutrition. Similarly, the impact of interventions aimed exclusively at increasing food security and improving malnutrition among street-involved youth may be limited without the incorporation of components aimed at reducing crystal methamphetamine use.

Studies have shown that supply reduction strategies in the United States aimed at disrupting small-scale producers of crystal methamphetamine within the country have largely failed to stem an increase in rates of crystal methamphetamine use [9]. Consequently, evidence-based interventions focussing on demand reduction and other adverse health sequelae of crystal methamphetamine use must be developed.

Although the ARYS study is not a random sample, all cohort studies of high-risk or marginalized populations generally suffer from this limitation since there are rarely registries from which to draw random samples. As well, our data was based on self-report and could therefore have resulted in socially desirable reporting [10], which may have consequently lowered reported rates of illicit drug use [11]. However, we know of no reason why risks of malnutrition would be differentially reported by methamphetamine users and non-users.

In summary, over one-fifth of our cohort reported being at risk of malnutrition in the previous six months as defined by not having enough money to buy food, and in multivariate analysis only non-injection crystal methamphetamine use was independently associated with this problem. This finding suggests that the impact of current health and preventive interventions aimed at addressing issues surrounding either crystal methamphetamine use or malnutrition among street-involved youth may be limited without taking into account the relationship between these health behaviours. Finally, further prospective and qualitative research is needed into the potential role of crystal methamphetamine use in mediating food security among this population, and prospective study will be required to examine the long term impact of crystal methamphetamine on nutrition-related health outcomes.

## Competing interests

DW, TK and EW have no conflicts of interest to declare. JM has received grants from, served as an ad hoc adviser to, or spoken at events sponsored by Abbott, Argos Therapeutics, Bioject Inc., Boehringer Ingelheim, BMS, Gilead Sciences, GlaxoSmithKline, Hoffmann-La Roche, Janssen-Ortho, Merck Frosst, Panacos, Pfizer Ltd., Schering, Serono Inc., TheraTechnologies, Tibotec (J&J), and Trimeris.

## Authors' contributions

DW and EW drafted the original manuscript and initiated the design of the study. RZ performed the statistical analyses. TK and JM participated in the design of the study and participated in substantial revisions of the manuscript. All authors read and approved the final manuscript.
